# One view or two views for wide-angle tomosynthesis with synthetic mammography in the assessment setting?

**DOI:** 10.1007/s00330-021-08079-2

**Published:** 2021-07-29

**Authors:** Paola Clauser, Pascal A. T. Baltzer, Panagiotis Kapetas, Ramona Woitek, Michael Weber, Federica Leone, Maria Bernathova, Thomas H. Helbich

**Affiliations:** 1 Department of Biomedical Imaging and Image-Guided Therapy, Medical University of Vienna/General Hospital Vienna, Waehringer Guertel 18-20, Vienna, Austria; 2 Division of General and Pediatric Radiology, Department of Biomedical Imaging and Image-Guided Therapy, Medical University of Vienna, Waehringer Guertel 18-20, 1090 Vienna, Austria; 3 Ospedale Luigi Sacco – Polo Universitario, via G.B. Grassi 74, 20157 Milan, Italy

**Keywords:** Breast cancer, Mammography, Digital breast tomosynthesis, 3D mammography

## Abstract

**Objectives:**

To evaluate the diagnostic performance in the assessment setting of three protocols: one-view wide-angle digital breast tomosynthesis (WA-DBT) with synthetic mammography (SM), two-view WA-DBT/SM, and two-view digital mammography (DM).

**Methods:**

Included in this retrospective study were patients who underwent bilateral two-view DM and WA-DBT. SM were reconstructed from the WA-DBT data. The standard of reference was histology and/or 2 years follow-up. Included were 205 women with 179 lesions (89 malignant, 90 benign). Four blinded readers randomly evaluated images to assess density, lesion type, and level of suspicion according to BI-RADS. Three protocols were evaluated: two-view DM, one-view (mediolateral oblique) WA-DBT/SM, and two-view WA-DBT/SM. Detection rate, sensitivity, specificity, and accuracy were calculated and compared using multivariate analysis. Reading time was assessed.

**Results:**

The detection rate was higher with two-view WA-DBT/SM (*p* = 0.063). Sensitivity was higher for two-view WA-DBT/SM compared to two-view DM (*p* = 0.001) and one-view WA-DBT/SM (*p* = 0.058). No significant differences in specificity were found. Accuracy was higher with both one-view WA-DBT/SM and two-view WA-DBT/SM compared to DM (*p* = 0.003 and > 0.001, respectively). Accuracy did not differ between one- and two-view WA-DBT/SM. Two-view WA-DBT/SM performed better for masses and asymmetries. Reading times were significantly longer when WA-DBT was evaluated.

**Conclusions:**

One-view and two-view WA-DBT/SM can achieve a higher diagnostic performance compared to two-view DM. The detection rate and sensitivity were highest with two-view WA-DBT/SM. Two-view WA-DBT/SM appears to be the most appropriate tool for the assessment of breast lesions.

**Key Points:**

*• Detection rate with two-view wide-angle digital breast tomosynthesis (WA-DBT) is significantly higher than with two-view digital mammography in the assessment setting.*

*• Diagnostic accuracy of one-view and two-view WA-DBT with synthetic mammography (SM) in the assessment setting is higher than that of two-view digital mammography.*

*• Compared to one-view WA-DBT with SM, two-view WA-DBT with SM seems to be the most appropriate tool for the assessment of breast lesions.*

## Introduction

Digital breast tomosynthesis (DBT) is emerging as the standard imaging modality for breast cancer diagnosis, based on improvements in both diagnostic and screening imaging outcomes [1–7]. Concerns over increased radiation dose [8, 9] have prompted the development of synthetic mammography (SM) in which two-dimensional images are reconstructed from the DBT data to replace the full-field digital mammography (DM) portion of the examination [10].

Image acquisition for DBT occurs with the x-ray tube moving in an arc, which varies, depending on the manufacturer, between 15° (narrow angle) and 50° (wide angle) [11]. In general, a wide-angle acquisition results in more tomographic information and yields a better vertical (z axis) resolution and delineation of soft tissues [12]. Based on the improved resolution of wide-angle systems, several studies have been performed that suggest the use of one-view (mediolateral oblique) wide-angle DBT (WA-DBT) only, with or without the second-view (cranio-caudal) DM [13–15]. The rationale is that the additional quasi-3D information of a one-view WA-DBT might obviate the need for a second view, which is currently also considered essential when using DBT. The one-view WA-DBT strategy would allow a further reduction of the radiation dose and add to the reduction already achieved with SM. In addition, this could lead to a reduction in reading time, as well as a reduction in radiologists’ fatigue when reading two-view DBT [16–18].

To test this hypothesis, we performed a retrospective study to evaluate the detection rate and diagnostic performance of one-view (mediolateral oblique) WA-DBT combined with SM, compared to a two-view WA-DBT combined with SM, and two-view DM alone in an assessment setting.

## Materials and methods

This retrospective study was approved by the ethics committee of our university. The need for written informed consent for the use of routine medical data records was waived. The study was performed in accordance with the Declaration of Helsinki statement for medical research involving human subjects.

Eligible subjects were patients who underwent two-view DM and WA-DBT because of inconclusive or suspicious findings seen during a screening examination on mammography and/or ultrasound (based on the Breast Imaging Reporting and Data System [BI-RADS] 0, 4, or 5 category) between March and June 2015. Women who presented with symptoms (palpable lumps, nipple discharge) and women who had follow-up exams after breast cancer treated with breast-conserving surgery were also included. The exclusion criterion to generate our study cohort was the absence of a standard of reference (image-guided biopsy, surgery, or at least 24 months follow-up in case of negative examinations (BI-RADS 1)). Further exclusion criteria were pregnant or lactating women, women undergoing neoadjuvant chemotherapy for a known breast cancer, and women who underwent mastectomy for a previous breast cancer.

This manuscript presents additional results from a dataset of patients, which has been published previously [19].

Included in the analysis were 205 women (mean age, 56.2 years; range, 36–84): 49 had no mammographic findings (BI-RADS 1), 135 had one lesion (BI-RADS 2–5), 19 women had two lesions, and two women had three lesions. The BI-RADS 1 cases were used as confounders to perform the detection task and were not considered in the further data analysis.

### Image acquisition

DM combined with WA-DBT was acquired with a commercially available system (Mammomat Inspiration, Siemens Healthineers), using the two standard views (cranio-caudal and mediolateral oblique) during the same breast compression. This device is characterized by a tungsten/rhodium anode/filter combination. The DBT view was acquired by 25 projections over an angular range of 50°. The DBT projections were reconstructed using EMPIRE technology (Siemens Healthineers), including statistical artifact reduction to mitigate out-of-plane artifacts and iterative filtering in image space to suppress noise. The resulting tomosynthesis slices have an in-plane resolution of 0.085 mm × 0.085 mm and are 1 mm apart. The DBT volumes SM (Insight 2D, Siemens Healthineers) were reconstructed based on a 3D volume ray-casting method in order to obtain the exact same distribution of calcifications and the same tissue structures as in the DMs.

The average acquisition time using this system is 1 s for one-view DM and below 25 s for one-view WA-DBT. Considering also the time for positioning and reconstruction, the estimated examination time for two-view DM, one-view WA-DBT with SM, and two-view WA-DBT with SM is below 4 min, 4 min, and 8 min respectively.

### Image analysis

Four radiologists with at least 7 years of experience in breast imaging performed the readings on a dedicated workstation (syngo.Breast Care, Siemens Healthineers) with high-resolution monitors. Prior to data collection, all readers analyzed a series of 20 test cases with SM in order to become familiar with typical image appearance. These reconstructed, synthesized 2D images were provided by Siemens Healthcare and were not part of the study.

Each reading session included all the 205 cases presented in a randomized order with different reading protocols. Each case was displayed only once per reading session. For this study, we considered three different reading protocols: two-view WA-DBT with SM, one-view (mediolateral oblique) WA-DBT with SM, and two-view DM. The mediolateral oblique view was chosen over the cranio-caudal view as it allows the evaluation of a larger area of breast parenchyma. The reading sessions were separated by a wash-out period of at least 3 weeks to avoid memory bias. Readers were aware of the inclusion criteria of the study, but they were blinded to the clinical history of the patients, previous and additional imaging (previous mammography, US or MRI), and histology.

During each reading session, readers were asked to define breast density using the classification suggested by BI-RADS [20], define the presence or absence of one or more lesions per breast (detection task), define lesion conspicuity using a scale from 1 (not conspicuous) to 10 (very conspicuous), define lesion type (mass, calcifications, architectural distortion, and asymmetry), and assign a BI-RADS category [20] to each detected lesion. Reading times were measured from the timepoint the case was available for review to the timepoint a BI-RADS category was assigned. Data on radiation exposure (average glandular dose, AGD) were collected. Lesion size was measured by a fifth reader, aware of lesion location and histology.

### Statistical analysis

All statistical computations were performed using IBM SPSS Statistics for Windows version 24.0.2. Detection rate, sensitivity, specificity, and accuracy were calculated for each reader. The detection rate was measured as the number of lesions detected of the total number of lesions included, both benign and malignant. Only the cases in which a lesion was present were included in the diagnostic performance analysis. Sensitivity was measured as the number of lesions assigned a BI-RADS 4 or 5 of the total number of malignant lesions. Undetected lesions were treated as false negatives. Specificity was measured as the number of lesions assigned a BI-RADS ≤ 3 on benign lesions detected by the reader. To compare the three protocols and the four readers in terms of lesion conspicuity, lesion detection, and diagnostic performance, generalized estimating equations (GEE) were used to obtain multiple measurements per case. A further multivariate analysis using GEE was performed to evaluate the effect of breast density and lesion type on the detection rate and diagnostic accuracy. To evaluate the effect of lesion size on the detection rate, lesions were divided into three groups (below 10 mm; 11–20 mm; above 21 mm) and the chi-square test for trends was used to analyze the effect of lesion size on detection rate. Fleiss’ kappa was used to assess inter-reader agreement for the BI-RADS category. Mixed-model analyses of variance were used to analyze reading times. A *p* value < 0.05 was considered statistically significant. For post hoc tests, Bonferroni corrections were used.

## Results

Included in the analysis were 205 women with 179 lesions (89 malignant and 90 benign). Details about lesion histology and types of lesion are summarized in Table 1. Mean lesion size measured on WA-DBT was 23 mm (standard deviation 15.6).

**Table 1:** Histology and lesion type of the lesions included in the analysis

Histology	Number of lesions (%)
Malignant	89 (49.7)
Invasive carcinoma NST	43 (48.3)
Invasive carcinoma NST with DCIS	29 (32.6)
Invasive lobular carcinoma	3 (3.4)
Invasive lobular carcinoma with DCIS	2 (2.2)
Ductal carcinoma in situ	11 (12.4)
Angiosarcoma	1 (1.1)
Benign	90 (50.3)
Fibrocystic changes	38 (42.2)
Fibroadenoma	21 (23.4)
Papilloma	17 (18.9)
Adenosis	4 (4.4)
Other	10 (11.1)
Lesion type	179 (100)
Mass	87 (48.6)
Microcalcifications	65 (36.3)
Architectural distortion	19 (10.6)
Asymmetry	8 (4.5)

*NST* non-special type, *DCIS* ductal carcinoma in situ, *Other* periductal mastitis, pseudoangiomatous stromal hyperplasia, hamartoma, inflammation

### Detection rate

Results are summarized in Table 2. Overall, two-view WA-DBT with SM achieved the highest detection rate (78.5%), followed by two-view DM (75.4%), and one-view WA-DBT with SM (75%). GEE showed that the detection rate was dependent on the reader (*p* < 0.001). Despite this, the detection rate was higher for two-view WA-DBT with SM for all four readers. These findings were confirmed at multivariate analysis also taking lesion type into account. The detection rate was not dependent on lesion type, though the detection rate for soft-tissue findings such as architectural distortions tended to be higher for two-view WA-DBT with SM (*p* = 0.073).

**Table 2:** Lesion detection rate for each reader for the three reading protocols: two-view digital mammography (2v-DM), one-view wide-angle digital breast tomosynthesis with synthetic mammography (1v-WA-DBT/SM), and two-view WA-DBT with SM (2v-WA-DBT/SM)

	2v-DM N° (%)	1v-WA-DBT/SM N° (%)	2v-WA-DBT/SM N° (%)
Detection			
Reader 1	138 (77.1)	135 (75.4)	139 (77.7)
Reader 2	123 (68.7)	120 (67.0)	131 (73.2)*
Reader 3	136 (76.0)	138 (77.1)	140 (78.2)
Reader 4	143 (79.9)	144 (80.4)	152 (84.9)*

*N°* number of lesions detected of the total of 179 lesions included in the analysis

*Significant difference compared to 2v-DM

The detection rate improved with increasing lesion size for all four readers when evaluating both DM (*p* ≤ 0.020) and with one-view WA-DBT with SM (*p* ≤ 0.041). The detection rate improved with increasing lesion size for only two of the four readers when evaluating two-view WA-DBT with SM (*p* ≤ 0.008 and *p* ≥ 0.404, respectively).

### Diagnostic performance

The BI-RADS categories assigned to the detected lesions by each reader with each reading protocol are summarized in Table 3.

**Table 3:** Distribution of the ACR BI-RADS categories in the detected lesions per reader and reading protocols (two-view digital mammography (2v-DM), one-view wide-angle digital breast tomosynthesis with synthetic mammography (1v-WA-DBT/SM), and two-view WA-DBT with SM (2v-WA-DBT/SM))

Reading protocol	Reader	BI-RADS 2	BI-RADS 3	BI-RADS 4	BI-RADS 5
2v-DM	1	40	9	53	36
	2	28	9	44	42
	3	32	21	50	33
	4	48	17	44	34
1v-WA-DBT/SM	1	40	17	40	38
	2	24	7	24	65
	3	25	28	37	48
	4	37	20	43	44
2v-WA-DBT/SM	1	43	11	43	42
	2	27	7	27	70
	3	31	22	34	53
	4	41	14	46	51

**Fig. 1:**
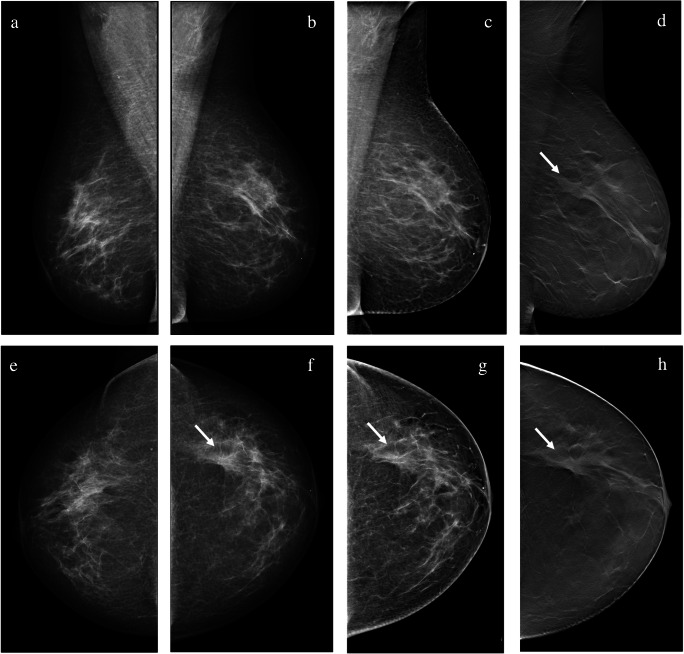
A 56-year-old woman with suspicious calcifications on the right side (benign at biopsy). An architectural distortion was visible in the upper outer quadrant of the left breast (arrow). The lesion was detected and correctly classified as suspicious by one in four readers with digital mammography (DM) and with one-view wide-angle digital breast tomosynthesis with synthetic mammography (WA-DBT with SM), while it was detected and classified as suspicious by three in four readers with two-view WA-DBT with SM. The lesion was biopsied under ultrasound guidance, and the diagnosis was invasive ductal carcinoma grade 2 with ductal carcinoma in situ (**a**, **b** DM bilateral mediolateral oblique view (MLO); **c** SM MLO; **d** DBT MLO; **e**, **f** DM bilateral cranio-caudal view (CC); **g** SM CC; **h** DBT CC)

Results are summarized in Table 4. The overall sensitivity of two-view WA-DBT with SM was 83.1%, and it was higher than the sensitivity of one-view WA-DBT with SM (79.8%, *p* = 0.058) and two-view DM (72.5%, *p* = 0.001). The differences in sensitivity between one-view WA-DBT with SM and two-view DM were not statistically significant (*p* = 0.226) (Fig. 1).

**Table 4:** Diagnostic performance for each reader for the three reading protocols: two-view digital mammography (2v-DM), one-view wide-angle digital breast tomosynthesis with synthetic mammography (1v-WA-DBT/SM), and two-view WA-DBT with SM (2v-WA-DBT/SM)

	2v-DM % (N°)	1v-WA-DBT/SM % (N°)	2v-WA-DBT/SM % (N°)
Sensitivity			
Reader 1	77.5 (69/89)	76.4 (68/89)	79.8 (71/89)
Reader 2	71.9 (64/89)	82.0 (73/89)	85.4* (76/89)
Reader 3	73.0 (65/89)	80.9 (72/89)	82.0 (73/89)
Reader 4	67.4 (60/89)	79.8 (71/89)	85.4* (76/89)
Specificity			
Reader 1	63.5 (40/63)	78.3 (47/60)	74.2 (46/62)
Reader 2	46.3 (25/54)	60.0 (27/45)	53.7 (29/54)
Reader 3	63.5 (40/63)	64.9 (37/57)	72.6 (45/62)
Reader 4	65.6 (42/64)	72.3 (47/65)	68.6 (48/70)
Accuracy			
Reader 1	71.7 (109/152)	77.2 (115/149)	77.5 (117/151)
Reader 2	62.2 (89/143)	74.6* (100/134)	73.4* (105/143)
Reader 3	69.1 (105/152)	74.7 (109/146)	78.1 (118/151)
Reader 4	66.7 (102/153)	76.6 (118/154)	78.0* (124/159)

*Significant difference compared to 2v-DM

**Fig. 2:**
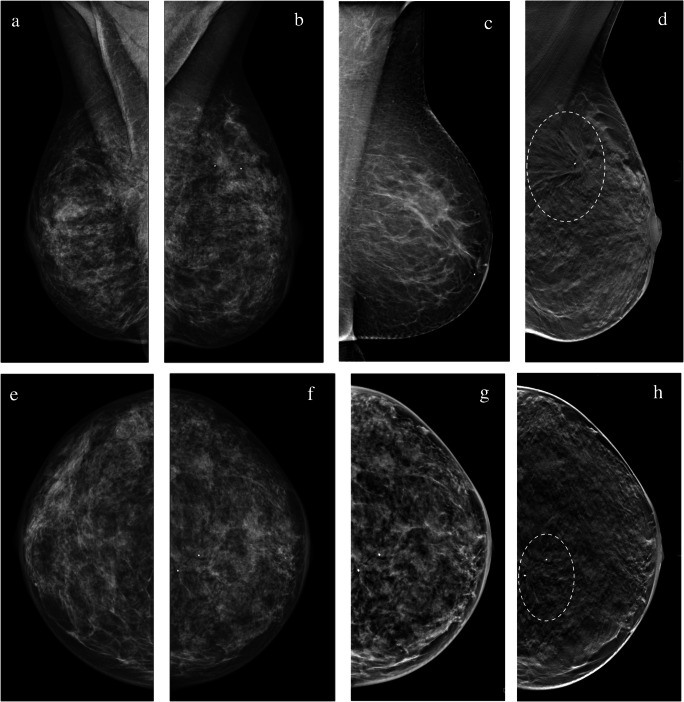
A 51-year-old woman with extremely dense breasts. An architectural distortion was visible in the upper quadrant of the left breast (circle). The lesion was detected and classified as probably benign on digital mammography (DM) by one in four readers and suspicious by the other three readers. All four readers agreed in identifying the lesion as suspicious when evaluating one-view wide-angle digital breast tomosynthesis with synthetic mammography (WA-DBT with SM) and two-view WA-DBT with SM. The lesion was biopsied under ultrasound guidance and the diagnosis was fibrotic breast tissue with multiple papillomas. The absence of malignancy was confirmed at surgery. (**a**, **b** DM bilateral mediolateral oblique view (MLO); **c** SM MLO; **d** DBT MLO; **e**, **f** DM bilateral cranio-caudal view (CC); **g** SM CC; **h** DBT CC)

Overall specificity ranged from 60.2% for two-view DM to 69.9% for one-view DBT with SM. Specificity was better for WA-DBT reading protocols, though the difference was not statistically significant compared to two-view DM (two-view DBT with SM, *p* = 0.104; one-view DBT with SM, *p* = 0.061) (Fig. 2).

The accuracy of two-view WA-DBT with SM and one-view WA-DBT did not differ significantly (overall 76.8% and 75.8%, respectively); however, both were significantly higher than two-view DM (67.5%) (two-view WA-DBT with SM: *p* < 0.001, one-view WA-DBT with SM: *p* = 0.003).

GEE showed that sensitivity and accuracy were dependent on the reading protocol used (both *p* < 0.001), but not on the reader (*p* = 0.162 and *p* = 0.334, respectively). In contrast, variations in specificity were not dependent on the reader (*p* = 0.063) or the reading protocol (*p* = 0.059).

Inter-reader agreement in the BI-RADS assessment was fair to moderate with all three reading modalities: 0.413 for DM, 0.383 for two-view WA-DBT with SM, and 0.404 for one-view WA-DBT with SM.

### Effect of breast density and lesion type

Accuracy decreased with increasing breast density for all three reading protocols and all four readers (Table 5). One-view and two-view WA-DBT with SM performed better than DM, regardless of breast density. Multivariate analysis showed that breast density significantly influenced the accuracy, regardless of the reading protocol (*p* = 0.002).

**Table 5:** Average accuracy of the three reading protocols for different breast densities, classified as according to the American College of Radiology (ACR) BI-RADS lexicon, and different lesion types

Accuracy	2v-DM % (95% CI)	1v-WA-DBT/SM % (95% CI)	2v-WA-DBT/SM % (95% CI)
ACR a	83.3 (71.1–91.1)	83.5 (70.0–91.7)	88.8 (76.2–95.1)
ACR b	79.3 (69.7–86.5)	84.1 (75.1–90.3)	87.5 (79.6–92.6)
ACR c	72.8 (64.7–79.6)	84.4 (77.1–89.6)	81.5 (74.9–86.7)
ACR d	60.6(44.9–74.4)	69.0(55.9–79.7)	66.7(50.8–79.5)
Mass	83.7 (77.3–88.6)	86.8 (80.6–91.2)	90.8 (85.5–94.3)
Microcalcifications	65.5 (53.8–75.5)	78.9 (68.1–86.7)	74.7 (63.3–83.5)
Architectural distortion	64.1 (39.9–82.8)	71.3 (42.7–89.2)	56.9 (36.5–75.2)
Asymmetry	40.7 (13.8–74.6)	61.0 (25.1–88.0)	72.5 (43.7–89.9)

*2v-DM* two-view digital mammography, *1v-WA-DBT/SM* one-view wide-angle digital breast tomosynthesis with synthetic mammography, *2v-WA-DBT/SM* two-view wide-angle digital breast tomosynthesis with synthetic mammography

Accuracy varied for different lesion types, and multivariate analysis showed that the reading protocols and lesion type significantly affected accuracy (*p* = 0.024). Overall accuracy was higher with two-view WA-DBT with SM for masses and asymmetries, and it was higher with one-view WA-DBT with SM for calcifications and architectural distortions. DM did not outperform WA-DBT for any of the lesion types (Table 5).

### Lesion conspicuity, reading times, and radiation dose

Average conspicuity assigned by the four readers was 9.26 for DM, 9.42 for one-view WA-DBT with SM, and 9.45 for two-view WA-DBT with SM. Lesion conspicuity for DM was significantly lower compared to both one-view and two-view WA-DBT with SM (*p* = 0.011 at multivariate analysis).

Average reading time for the four readers was 48.1 s for DM, 63.2 s for one-view WA-DBT with SM, and 75.1 s for two-view WA-DBT with SM. Reading times for DM were significantly lower compared to one-view and two-view WA-DBT with SM (*p* < 0.001). Reading times for one-view WA-DBT with SM were significantly lower than for two-view WA-DBT with SM (*p* < 0.001).

The median radiation dose of the DM views was 1.04 mGy (95% CI, 0.58–2.28 mGy). As the dose factor for the WA-DBT views was set to 2.0, this resulted in a similar dose per breast for the two-view DM and the one-view WA-DBT with SM (2.08 mGy; 95% CI, 1.16–4.56 mGy). Two-view WA-DBT with SM had a twofold increased dose compared to the other two protocols (4.16 mGy; 95% CI 2.32–9.12 mGy).

## Discussion

The results of our study demonstrate that, in an assessment setting, two-view WA-DBT with SM achieves a higher detection rate and diagnostic performance compared to two-view DM. One-view WA-DBT with SM had a higher diagnostic accuracy compared to two-view DM, but there was no improvement in the detection rate. Furthermore, two-view WA-DBT with SM had a higher detection rate, but no significant improvement in diagnostic accuracy compared to one-view WA-DBT with SM.

The addition of two-view DBT to two-view DM allows for a significant increase in lesion detection rate [6, 21–24], but results in a relevant increase in radiation exposure [8, 9]. The availability of SM eliminates the need to acquire DM, consequently reducing the radiation exposure, while maintaining a detection rate comparable to that of two-view DM with DBT [6, 19, 25]. To further reduce radiation exposure, some studies have suggested using WA-DBT only in the mediolateral oblique view. Zackrisson et al [13, 15] showed that one-view WA-DBT with or without the addition of one-view (cranio-caudal) DM can outperform two-view DM in the screening setting. Similar results were found by Rodriguez-Ruiz et al [14] in a cancer-enriched population. SM was not available for the analysis in any of these studies. The use of one-view WA-DBT with SM could reduce the radiation dose by more than 50% compared to two-view DBT with DM [9, 19].

In our analysis, we found a marginal improvement in accuracy when evaluating one-view WA-DBT with SM compared to two-view DM, and a more evident improvement when using two-view WA-DBT with SM. The detection rate, sensitivity, and accuracy improved when two-view WA-DBT and SM were available. Our results are in-line with a review that analyzed studies comparing one-view and two-view DBT with two-view DM, and underlined that the improvements were more substantial and conspicuous when two-view DBT was available [22]. In this analysis [22], one-view DBT with SM was not considered, but our results indicate that the same conclusions reached for one-view and two-view DBT with DM are also applicable to one-view and two-view DBT with SM.

Overall, it seems that two-view DBT is more relevant in an assessment setting, where it is crucial to upgrade or downgrade a previous finding and to detect multiple lesions. In a screening setting, one-view WA-DBT protocols seem to be sufficient to significantly increase the cancer detection rate and improve the screening performance, compared to DM alone [13, 15]. In clinical practice, the evidence suggests that one-view WA-DBT (mediolateral oblique) should be preferred in screening, as it allows a reduction in reading times and radiation dose, and the second view (cranio-caudal) should be always performed in those patients recalled after screening or who present with symptoms, as it allows an additional improvement in detection and diagnostic performance [26–29].

Specificity did not differ significantly between the three reading protocols. This is also in agreement with previous European studies, which showed a limited effect of DBT on specificity and false-positive rates [14, 15, 23]. In addition, a recent meta-analysis underlined that, while the evidence suggests that DBT can increase the cancer detection rate in screening, no significant reduction in recall rate can be found when considering the overall data [25]. The use of SM rather than DM could also negatively influence specificity: previous analyses have indicated that the difficulties in the interpretation of findings in the synthetic images might lead to an increase in false positives [10, 30].

WA-DBT improved the performance particularly for breasts with scattered fibroglandular tissue (ACR b) and heterogeneously dense breasts (ACR c), while the improvement for fatty breasts (ACR a) and extremely dense breasts (ACR d) was only marginal. This is in agreement with the results by Zackrisson et al [15] using one-view WA-DBT, and with other studies performed with two-view narrow-angle DBT [3, 31], which also found that DBT was particularly relevant in intermediate breast densities (ACR b and c).

The availability of WA-DBT improved the accuracy for all lesion types, and the availability of two-view was particularly useful for the characterization of masses and asymmetries. This is in agreement with the literature and underlines that the quasi-3D information available from DBT significantly improves the evaluation of soft-tissue lesions [26, 27]. DBT, with and without SM, also seems to ensure a performance at least comparable to DM for calcifications [32–34], although, particularly for calcifications, studies have suggested that the use of SM might lead to a higher rate of false-positive findings [30, 35, 36].

Inter-reader agreement for BI-RADS was fair to moderate with all imaging modalities. This is in-line with previous published works that showed moderate inter-reader agreement [37, 38]. As opposed to Galati et al [39], we did not find an improvement in the inter-reader agreement when DBT was available.

Lesion conspicuity was rated as very good for all imaging modalities, but it was significantly higher when WA-DBT was available. Both Mariscotti et al [29] and Murakami et al [40] have already shown that lesion conspicuity is comparable between SM and DM. Of note, SM can improve lesion visibility in dense breasts and for calcified lesions [40].

We found, consistent with the published literature [16], a significant increase in reading times when WA-DBT was evaluated. A reduction in reading time and, consequently, in radiologists’ fatigue is essential in a screening setting, where high volumes of examinations have to be evaluated in a short period of time [16–18]. The increase in reading times can be acceptable in an assessment setting, particularly when considering the advantages of improved lesion detection and characterization.

Our study has some limitations. First, it is a retrospective study, performed in a single assessment center. As the only exclusion criterion applied was the absence of a standard of reference (histology or 2 years’ follow-up), we believe our dataset reflects the reality of an assessment center. No inference regarding the usefulness of one-view WA-DBT in a screening setting should be made based on our results. Only 19 patients presented with multiple lesions, and, of these, only six with multiple malignant lesions. Thus, it was not possible to perform a sub-analysis on the added value of WA-DBT for the evaluation of multifocal or multicentric lesions.

In conclusion, one-view and two-view WA-DBT with SM can achieve a higher diagnostic performance compared to two-view DM in an assessment setting. The detection rate and sensitivity were highest with two-view WA-DBT with SM. In the assessment of breast lesions, two-view WA-DBT with SM should become a standard and should not be replaced by one-view WA-DBT with SM or two-view DM.

## Abbreviations

ACR: American College of Radiology
BI-RADS: Breast Imaging Reporting and Data System
DM: Digital mammography
GEE: Generalized estimating equations
SM: Synthetic mammography
WA-DBT: Wide-angle digital breast tomosynthesis

## Declarations

### Guarantor

The scientific guarantor of this publication is Thomas H. Helbich.

### Statistics and biometry

One of the authors (M.W.) has significant statistical expertise.

### Study subjects or cohorts overlap

The study cohort has been previously reported in Clauser P., et al, Investigative Radiology 2019 54(2); 83–88. 10.1097/RLI.0000000000000513.

### Methodology

• retrospective

• cross-sectional study

• performed at one institution
